# Nesfatin-1/NUCB2 as a Potential New Element of Sleep Regulation in Rats

**DOI:** 10.1371/journal.pone.0059809

**Published:** 2013-04-01

**Authors:** Szilvia Vas, Csaba Ádori, Katalin Könczöl, Zita Kátai, Dorottya Pap, Rege S. Papp, György Bagdy, Miklós Palkovits, Zsuzsanna E. Tóth

**Affiliations:** 1 Department of Pharmacodynamics, Semmelweis University, Budapest, Hungary; 2 Department of Neuroscience, Karolinska Institute, Stockholm, Sweden; 3 Neuromorphological and Neuroendocrine Research Laboratory, Department of Anatomy, Histology and Embryology, Semmelweis University and the Hungarian Academy of Sciences, Budapest, Hungary; 4 Group of Neurochemistry, Hungarian Academy of Sciences, Budapest, Hungary; 5 Group of Neuropsychopharmacology, Hungarian Academy of Sciences, Budapest, Hungary; Karolinska Institutet, Sweden

## Abstract

**Study Objectives:**

Millions suffer from sleep disorders that often accompany severe illnesses such as major depression; a leading psychiatric disorder characterized by appetite and rapid eye movement sleep (REMS) abnormalities. Melanin-concentrating hormone (MCH) and nesfatin-1/NUCB2 (nesfatin) are strongly co - expressed in the hypothalamus and are involved both in food intake regulation and depression. Since MCH was recognized earlier as a hypnogenic factor, we analyzed the potential role of nesfatin on vigilance.

**Design:**

We subjected rats to a 72 h-long REMS deprivation using the classic flower pot method, followed by a 3 h-long ‘rebound sleep’. Nesfatin mRNA and protein expressions as well as neuronal activity (Fos) were measured by quantitative *in situ* hybridization technique, ELISA and immunohistochemistry, respectively, in ‘deprived’ and ‘rebound’ groups, relative to controls sacrificed at the same time. We also analyzed electroencephalogram of rats treated by intracerebroventricularly administered nesfatin-1, or saline.

**Results:**

REMS deprivation downregulated the expression of nesfatin (mRNA and protein), however, enhanced REMS during ‘rebound’ reversed this to control levels. Additionally, increased transcriptional activity (Fos) was demonstrated in nesfatin neurons during ‘rebound’. Centrally administered nesfatin-1 at light on reduced REMS and intermediate stage of sleep, while increased passive wake for several hours and also caused a short-term increase in light slow wave sleep.

**Conclusions:**

The data designate nesfatin as a potential new factor in sleep regulation, which fact can also be relevant in the better understanding of the role of nesfatin in the pathomechanism of depression.

## Introduction

Nesfatin-1, the N-terminal fragment of the nucleobindin2 protein (NUCB2) is a potent anorexigen decreasing nocturnal food intake in rodents in a dose-dependent manner [1]. Higher plasma nesfatin-1/NUCB2 (nesfatin) levels in overweight patients point to its role in food intake regulation also in humans [2]. In addition, nesfatin has been associated with further functions too, like processing emotional states, such as anxiety and stress [3], [4]. Since depression, a major cause of morbidity worldwide is also characterized by marked alterations in emotional states and feeding, initial research on the role of nesfatin regarding this field may have high relevance. As already has been established, patients with major depressive disorder possess high plasma level of nesfatin [5]. For plasma and cerebrospinal fluid nesfatin levels positively correlate, CNS problems related to alterations in nesfatin expression may underlie this elevation [2]. This is also supported by the fact that nesfatin mRNA content is elevated in the Edinger-Westphal nucleus of depressed male suicide victims [6]. Besides emotional and feeding disturbances, sleep-wake regulation is another function typically affected in depression [7]. Impaired sleep continuity, decreased rapid eye movement sleep (REMS) latency and elevated time spent in REMS are characteristic sleep-EEG changes in depressed patients [8], [9] moreover, antidepressant medication also alters sleep. As a consequence, the existence of a relationship between nesfatin and sleep regulation can be assumed [10].

The largest population of nesfatin neurons in the CNS can be found in the perifornical and lateral hypothalamic (LH) areas that belong to the dorsolateral hypothalamus (DLH) and in the zona incerta (ZI) [11], [12]. These areas are closely associated with sleep-wake regulation [13], and are also related to both control of feeding and depression [14]–[17]. Two main types of neurons forming intermingled, but separate populations were identified in this region before; the orexin and the melanin-concentrating hormone (MCH) producing cell groups, both increasing food intake, but acting oppositely on vigilance [18], [19]. Nesfatin is highly co - expressed with MCH, although a smaller portion of nesfatin neurons is MCH - negative [10]. Since MCH increases REMS [20], but unlike nesfatin, it is an orexigenic peptide, the possible role of nesfatin-1 in the regulation of sleep has a special interest.

Based on the facts above, in this study, we aimed to investigate the effect of intracerebroventricularly (icv) administered nesfatin-1 on vigilance stages, like wakefulness, REMS and non-REMS. We also raised the questions, whether selective REMS pressure impacts expression of nesfatin, and alters activation of the nesfatin - immunoreactive neurons in the DLH and the ZI.

To elucidate this, we performed REMS deprivation using the classic flower pot method [20], followed or not by rebound sleep, and determined nesfatin protein and mRNA expressions by ELISA and quantitative *in situ* hybridization (ISH) method. Finally, we analyzed the activation pattern of MCH - positive and - negative nesfatin cell populations under the different experimental conditions using Fos/nesfatin/MCH triple immunostainings.

## Materials and Methods

### Ethics Statement

Experiments were performed according to the European Communities Council Directive of 24 November 1986 (86/609/EEC) and the National Institutes of Health “Principles of Laboratory Animal Care” (NIH Publications No. 85-23, revised 1985), as well as specific national laws (the Hungarian Governmental Regulations on animal studies, December 31, 1998). All experiments were approved by the National Scientific Ethical Committee on Animal Experimentation, and permitted by the governement (Food Chain Safety and Animal Health Directorate of the Central Agricultural Office, Permit Number: 22.1/1375/7/2010). All surgery was performed under anesthesia, and all efforts were made to minimize suffering.

#### Animals

Male Wistar rats (Semmelweis University, Budapest, Hungary) weighing 300–350 g were used for the studies. Animals were kept with light-dark cycle of 12∶12 h (light on at 10∶00 and off at 22∶00, daylight type fluorescent tubes, 18 W, approximately 300 lx) at room temperature (21±1°C), and had free access to standard rodent chow and tap water. Animals were habituated to the conditions in the experimental room at least for two weeks.

### Experiment 1. Effects of REMS Deprivation and the ‘Rebound Sleep’ on the Expression of Nesfatin mRNA and Protein

#### REMS deprivation

REMS deprivation was performed using the flower pot method, as described earlier [20], [21]. Briefly, animals were placed on round platforms situated in the middle of a round water tank with a surface 0.5 cm above the water level at lights on (10∶00) for 72 h. The diameter of the platforms was either small (6.5 cm) or large (13 cm). As muscle atony is typical for REMS, animals on the small pots fall into the water immediately after entering REMS. Contrarily, rats on the large pots fit better on the surface and may sleep REMS, therefore they are used as stress controls. According to this, animals were randomly divided into six groups. The 1^st^ group; small (SP), and the 2^nd^ group; large pot (LP) kept rats, that were sacrificed after spent 72 h on the platforms. The 3^rd^ group; SP plus rebound (SPR), and the 4^th^ group; LP plus rebound (LPR) rats that, after spending 72 h on SP or LP platforms, respectively, were transferred to their home cages at lights on for 3h rebound sleep after which they were killed. The 5^th^ group; home cage (HC) and the 6^th^ group; HCR animals were controls, kept undisturbed in their home cages, and killed at the end of the experiment either at lights on (HC) or 3 h later (HCR), respectively. Rats were sacrificed by decapitation (n = 7 for ELISA, n = 9 for ISH) or perfused with 4% paraformaldehyde in 0.1 M phosphate buffered saline, pH = 7.4 (PBS) for immunohistochemistry (n = 5). The brains were removed and frozen on dry ice. Fixed tissue was cryoprotected in 20% sucrose overnight before freezing.

Rats on the platforms were fed *ad libitum* without restriction using a waterproof food supplier unit at a distance easy to approach. Body weight change and food intake of rats during the time spent on the platforms were measured.

#### *In situ* hybridization technique

The rat nesfatin-1 cDNA (246 bp) was purchased from Invitrogen (Budapest, Hungary, GenBank Acc: DY314804 ), cloned into a pBC KS+ vector and verified by sequencing. The rat corticotropin - releasing hormone (CRH) cDNA (468 bp) was kindly provided by W.S. Young 3^rd^, and used as earlier described [22]. The [35S]UTP-labeled sense and antisense riboprobes were prepared by *in vitro* transcription (Maxiscript KIT), according to the manufacturer’s protocol.

Hypothalamic regions of fresh frozen brains were cut into 12 µm thick serial coronal sections in a cryostat (Leica Microsystems GmbH, Wetzlar, Germany). The sections were thaw-mounted and air-dried at 37°C onto positively charged Superfrost Plus slides (Thermo Scientific, Budapest, Hungary). Slides were stored at −80°C until used. Hybridizations were performed overnight in humid chambers at 55°C with 10^6^ cpm/slide of the [35S]UTP-labeled probes, as described earlier [4]. After this step, sections were apposed to a BAS-MS imaging plate (Fuji Photo Film Co., LTD., Kanagawa, Japan, NJ) for 3 days, and then data were read out by a Fujifilm FLA-8000 Image Analyzer. Expression levels were evaluated from the images recorded on the phosphor imager. Mean grey values of the area of interest were measured by using the Image J 1.32j software (Wayne Rasband; NIH, Bethesda, MD, USA) on both sides of 4–6 sections in each animal. Background values measured in parallel were subtracted. The average/animal data were used for statistical evaluation.

#### ELISA measurements

Hypothalamic regions of fresh frozen brains were cut into 300 µm thick serial coronal sections in a cryostat (Leica). DLH with ZI was dissected by micropunch technique [23], using a special punching needle (inner diameter of 500 µm). Tissue pellets were stored at −80°C until further processing. Samples were homogenized in 200 µl of 0.1 M HCl/0.3 µM aprotinin solution (Sigma-Aldrich, Budapest, Hungary) by ultrasound sonication for 3×15 seconds on ice. Then, samples were centrifuged for 20 min at 15,300 rpm at 4°C. The supernatants were divided into two portions and dried by a vacuum concentrator (Savant Instruments Inc, Holbrook, NY, USA). The first portion was reconstituted in 40 µl 0.1 M Tris buffer (pH = 8.0) and the total protein concentration was determined by using a Lowry-based assay. The second portion was reconstituted in 400 µl 1x sample buffer provided in the ELISA kit and used for determining nesfatin protein concentrations, by a commercially available ELISA kit (Phonix Europe GmbH, Karlsruhe, Germany) according to the manufacturer’s protocol.

#### Immunohistochemistry

Hypothalami were cut into 50 µm thick serial coronal sections on a frigomobil (Frigomobil, Reichter-Jung, Vienna, Austria). Immunostaining started with blocking the endogenous peroxidase activity, using a 3% H_2_O_2_ solution (Sigma-Aldrich) for 15 min. Then, sections were blocked in 1% BSA and 0.5% TritonX-100/PBS all from Sigma-Aldrich for 1 h. The same solution was used to dilute the antibodies. Incubations were made for 2 days at 4°C in primary antibodies and for 1 h at room temperature, in secondary or tertiary antibodies. Sections were washed 3 times for 5 min in PBS following each incubation step. To block peroxidase enzyme used for visualization previously, and to prevent species cross-reactions caused by primary antibodies raised in the same hosts, sections were microwave-treated in 0.1 M citric-acid (pH = 6.0) for 5 min after each immunostaining [24].

Fos immunostaining was performed using rabbit anti-Fos primary antibody (1∶30,000, Santa Cruz Biotechnology, Inc., Santa Cruz, CA, USA) and anti-rabbit IgG polymer-HRP (Millipore, Budapest, Hungary) secondary antibody. The immunostaining was visualized by FITC-conjugated tyramide (Invitrogen, Budapest, Hungary). Next, sections were incubated in rabbit anti-nesfatin (1∶24,000, Phoenix Pharmaceuticals, Inc., Burlingame, CA, USA) and again in anti-rabbit IgG polymer-HRP (Millipore). The second immunostaining was developed by tyramide-conjugated Alexa Fluor 568 (Invitrogen). In case of triple immunostainings, rabbit anti-MCH was applied (1∶10,000, Phoenix Europe GmbH), followed by incubation in biotinylated anti-rabbit IgG as secondary antibody (1∶1,000, Vector Laboratories, Inc., Burlingame, CA, USA) and in extravidine-peroxidase (1∶1,000, Sigma). The MCH antigen was visualized by tyramide-conjugated biotin (Invitrogen) and Streptavidin-Cy5 (1∶1000, Jackson ImmunoResearch Europe Ltd, Newmarket, Suffolk, UK). Sections were mounted on non-coated slides, air - dried and coverslipped with DPX (Sigma).

#### Image analysis

Nesfatin - positive cell populations were analyzed in three areas: 1) ZI, 2) LH, 3) perifornical area, including the perifornical nucleus itself [25]. Images were captured bilaterally using a 20X objective (S Fluor 20X/0.75, ∞/0.17, WD1.0) on 3–5 sections per animal by a Nicon Eclipse E800 microscope attached to a Bio-Rad Radiance 2100 Rainbow confocal scanning system by sequential scanning. Cell counts and determination of co - localization were made using the AnalySIS Pro 3.2 program (Olympus, Soft Imaging Solutions GmbH, Münster, Germany), by simultaneous examination of the greyscale images of the separated channels and the colored overlay picture. To identify neurons in the pictures, a numbered grid of the same size was placed over the overlay picture and the greyscale pictures of the separated channels. Only neurons with visible cell nuclei were counted. Percentages of nesfatin/MCH double labeled neurons among the nesfatin positive ones, the percentages of Fos/nesfatin within the single nesfatin and Fos/nesfatin/MCH within the nesfatin/MCH double positive subpopulations were calculated per animal. Data are presented as the average of the results from 5 animals per group.

### Experiment 2. Effects of Exogenously Administered Nesfatin-1 on Vigilance

#### EEG measurements

Rats (n = 6) were equipped with electroencephalographic (EEG) and electromyographic (EMG) electrodes for EEG recordings, as described earlier [26], [27]. Stereotaxic surgery was performed under 2% halothane anesthesia (using Fluotec 3 halothane vaporizer). All efforts were made to minimize suffering of the animals. Briefly, stainless steel screw electrodes were implanted epidurally over the left frontal motor cortex (coordinates: anterior-posterior (A-P): 2.0 mm from bregma, lateral (L): 2.0 mm to the midline, [25] the left parietal cortex (A-P: 2.0 mm from lambda, L: 2.0 mm) for fronto-parietal EEG recordings, and a ground electrode was placed over the cerebellum. In addition, EMG electrodes (stainless steel spring electrodes embedded in silicon rubber, Plastics One Inc., Roanoke, VA, USA) were placed into the muscles of the neck. At the same time, a plastic cannula was implanted into the right lateral ventricle (coordinates: A-P: −0.8 mm to the bregma level, L: 2.0 mm, and ventral: 4.0 mm below the skull surface). The cannula and the EEG electrodes were anchored to the skull with dental cement (SpofaDental a.s., Markova, Czeh Republic). A stainless steel obturator was inserted into the guide cannula and was kept patent until use. After surgery, rats were kept in single cages in the recording chamber, and were allowed to recover for 7 days. Animals were then habituated to the recording conditions for five days before experiment started by attaching them to the polygraph using a flexible recording cable and an electric swivel, fixed above the cages, permitting free movements. During the recovery and the habituation period to the recording conditions, animals were also handled daily to minimize future experimental stress, as described earlier [28]. On the day of the experiment, 25 pmol/5 µl of nesfatin-1 dissolved in physiological saline was injected into the lateral ventricle of rats at light onset [29]. Control rats received 5 µl physiological saline icv. After injections, animals returned to their home cages and vigilance was recorded for 24 h. The placement of the cannula was verified at the end of the study by injecting 10 nM/3 µl of angiotensin II icv. Only animals reacting with an intensive drinking response were included in the study.

#### Sleep scoring

The vigilance states were classified by SleepSign for Animal sleep analysis software (Kissei Comtec America, Inc., USA) for 4 sec periods using conventional criteria [27], [30] followed by visual supervision of an expert scorer who was blind to experimental treatment.

The differentiated vigilance states were the following: 1) wakefulness; EEG is characterized by low amplitude activity at beta (14–30 Hz), alpha (8–13 Hz) and theta (5–9 Hz) frequencies accompanied by high or low EMG and motor activity, 2) REMS; low amplitude and high frequency EEG activity with regular theta waves (5–9 Hz) accompanied by silent EMG and motor activity with occasional twitching, 3) intermediate stage of sleep (IS); a brief stage just prior to REMS and sometimes just after it, characterized by unusual association of high amplitude spindles (mean 12.5 Hz) and low-frequency (mean 5.4 Hz) theta rhythm; 4) non-REMS; slow cortical waves (0.5–4 Hz) accompanied by reduced EMG and motor activity [27]. In sleep analysis after icv nesfatin-1 treatment, the following vigilance parameters were calculated: time spent in active (AW) and passive (PW) wake, REMS, IS, as well as in light slow wave sleep (SWS1) and deep slow wave sleep (SWS2), per hour. Additionally, specific parameters were calculated, namely, the number and the average duration of episodes in REMS, IS, SWS1 and SWS2. An episode of any vigilance stages was defined as an item lasting at least 4 sec and not interrupted by any other vigilance stages for longer than 4 sec. Sleep fragmentation was defined as the number of wake epochs (AW, PW) after a sleep stage (SWS1, SWS2, REM, IS). Since despite the habituation procedure that minimized stress, the process of icv administration disturbed daily rhythm of the animals, the 1^st^ h of all EEG recordings had been omitted from the evaluation and sleep scoring, and the analysis was performed from the beginning of the 2^nd^ h to the end of the 6^th^ h of passive phase.

#### Statistics

We used STATISTICA 7.0 program (Statsoft Inc., Tulsa, OK, USA) to statistical analysis. Two-Way ANOVA followed by all pairwise comparisons with Student-Newman-Keuls Method was used for determining significance in nesfatin-1 mRNA and protein expressions and CRH mRNA expression. One Way ANOVA and One Way ANOVA on Ranks followed by all pairwise comparisons with Tukey or Dunn’s Method was applied to analyze morphological data as well as cumulative food intake and body weight change. Sleep data of different vigilance stages, summarized hourly, were evaluated by Two Way ANOVA for repeated measures (repeated factor: time). In case of inhomogeneous variances of the experimental groups, repeated measures ANOVA on Ranks was performed. For *post hoc* analysis, all pairwise comparisons with Tukey HSD were used. The results are presented as mean ± standard error of mean (SEM).

## Results

### Changes in Nesfatin mRNA and Protein Levels after REMS Deprivation and Rebound

**Figure 1:**
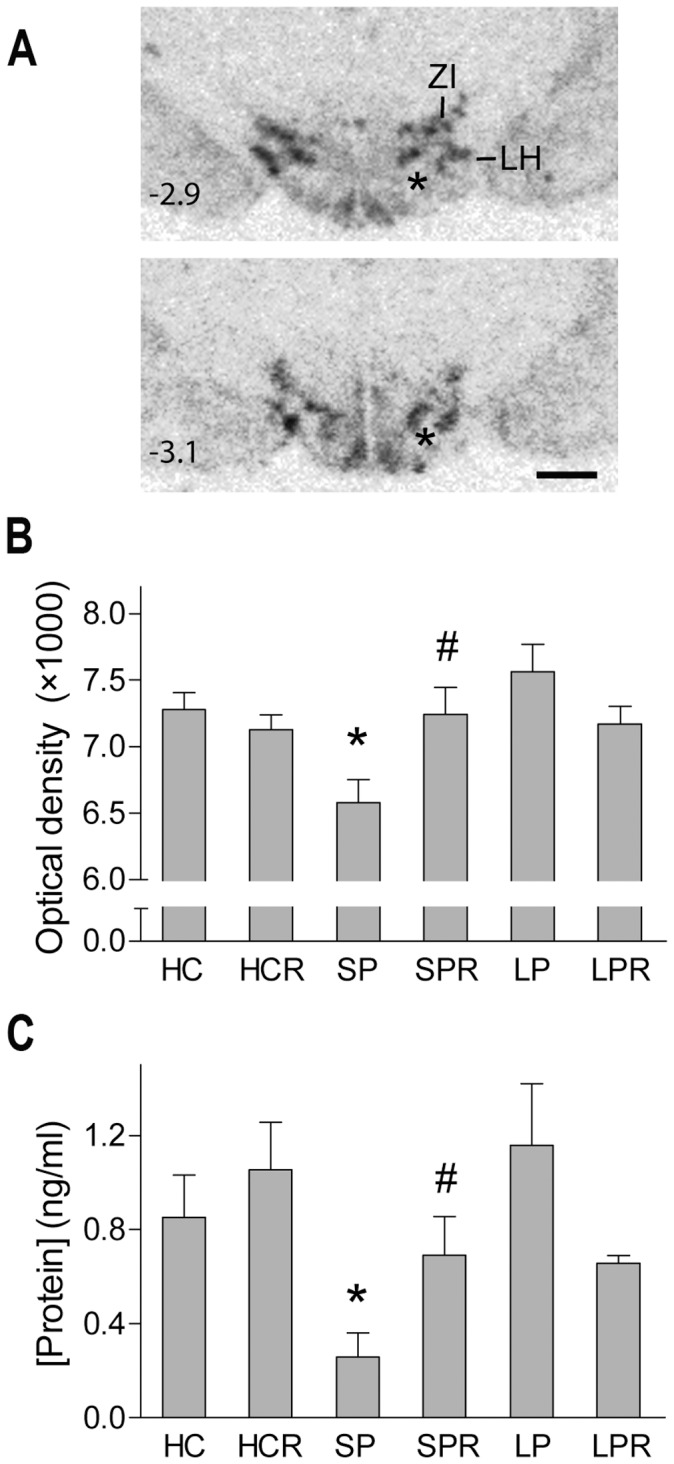
Nesfatin-1/NUCB2 expression as controlled by rapid eye movement sleep stage. A . Autoradiographic images of coronal sections through the middle portion of the hypothalamus showing the area of interest hybridized against nesfatin-1/NUCB2 mRNA in control animals. The upper and the lower panels show two different rostro - caudal levels. Distance from the bregma is indicated at bottom left in millimeters. Asterix: fornix, LH: lateral hypothalamic area, ZI: zona incerta, scale: 1 mm. **B**,**C**. Nesfatin-1/NUCB2 mRNA and protein levels in the different experimental groups determined by quantitative ISH and ELISA measurements, respectively. HC: home cage control, sacrificed with the animals kept on platforms, HCR: home cage control “rebound”, sacrificed at the same time as rebound groups, SP: small pot, SPR: small pot plus sleep rebound, LP: large pot, LPR: large pot plus sleep rebound groups. p*<0.05 *vs.* all other groups, p# <0.05 *vs.* SP group, n = 5–9 for **B** and n = 4–7 for **C**.

The DLH with the ZI hosts the most prominent nesfatin - expressing cell population in the hypothalamus regarding the number of the cells and the expression level of the peptide (Fig. 1A). Nesfatin mRNA and protein expressions have responded for the experimental paradigms with parallel changes. There was no difference between HC and HCR groups, indicating that there is little or no spontaneous switch in nesfatin expression within three hours after the lights on (Fig. 1B,C). Nevertheless, a significant interaction was seen between the type of the platform and level of the rebound (nesfatin mRNA and protein measurements, pot x rebound interaction: F_(2,39)_ = 4.90, p<0.05 and F_(2,28)_ = 4.10, p<0.05, respectively). Both nesfatin mRNA and protein levels decreased exclusively in REMS - deprived small pot kept animals, followed by a relative increase after the three hour rebound sleep (Fig. 1B,C). Large pot conditions (with or without rebound) did not affect nesfatin expression (Fig. 1B,C).

### Stress - Related Changes in Rats following REMS Deprivation

**Figure 2:**
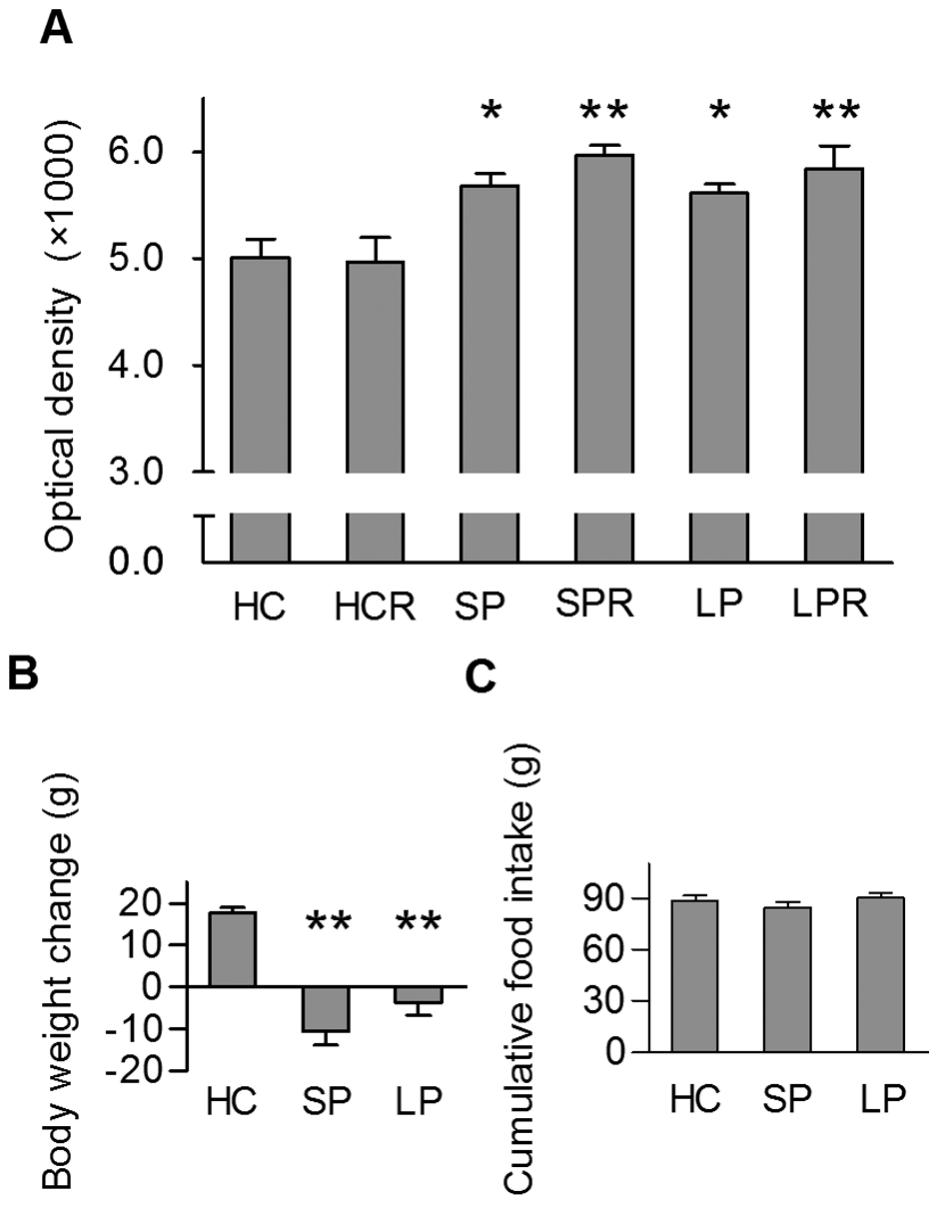
Stress-related changes and energy balance of the experimental animals. Data show significantly increased levels of CRH mRNA in the hypothalamic paraventricular nucleus (**A**) and decreased body weights (**B**) both in the SP and LP kept animals, compared to HC, without difference between the above mentioned groups. **C**. Cumulative food intake shows no alterations. HC: home cage control, sacrificed with the animals kept on platforms, HCR: home cage control “rebound”, sacrificed at the same time as rebound groups, SP: small pot, SPR: small pot plus sleep rebound, LP: large pot, LPR: large pot plus sleep rebound groups. Data are shown as mean ± SEM, n = 10–11, p*<0.05, p**<0.01 *vs*. HC.

CRH mRNA level in the hypothalamic paraventricular nucleus, the centre of the stress response in the central nervous system, as well as body weight changes and cumulative food intake, during the 72 h spent on the platforms, were measured. Both SP and LP kept animals showed elevated CRH mRNA levels and a negative energy balance, compared to HC controls, indicating stress evoked by the experimental conditions. However, there was no significant difference between the LP and SP kept rats regarding these parameters (Fig. 2A,B). Cumulative food intake did not change between the groups (Fig. 2C), confirming that negative energy balance observed was not caused by fasting on platforms. However, animals on either type of platform spent considerable amount of time with swimming (unpublished data), thus fur of the animals are often wet, and this fact can also contribute to the increased energy expenditure of LP and SP kept rats.

### Activation of Nesfatin - Positive Neuronal Cell Population

**Figure 3:**
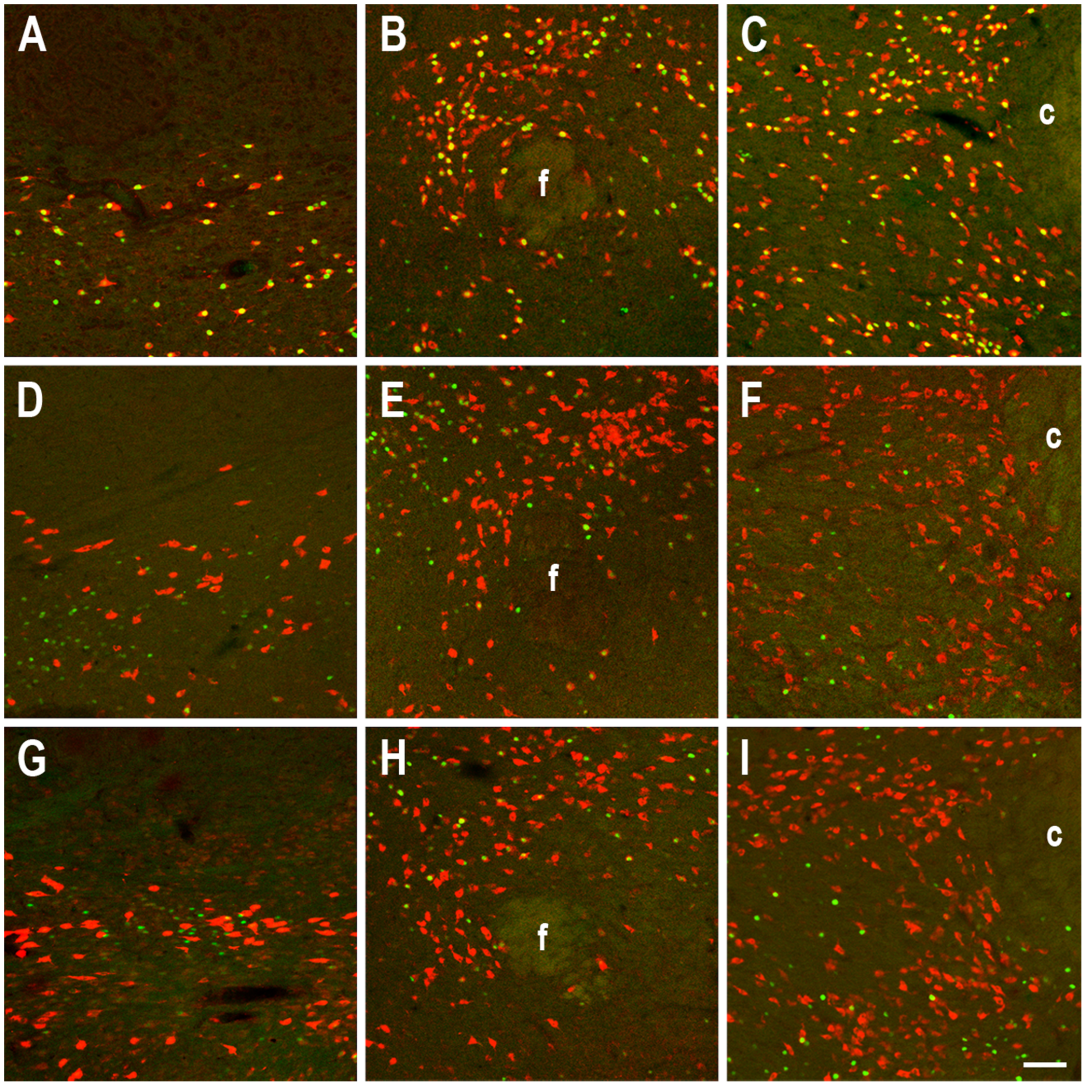
Effect of rebound sleep on the activity of nesfatin1/NUCB2 (nesfatin) - positive cell population. Fos (green) and nesfatin (red) double fluorescent immunostainings showing the three investigated areas arranged in columns (left: zona incerta, middle: perifornical area, right: lateral hypothalamic area) in rapid eye movement sleep (REMS) - deprived - sleep rebound (**A–C**), REMS - deprived without rebound (**D–F**) and home cage kept (**G–I**) animals. c: capsula interna, f: fornix, scale: 100 µm.

Since REMS deprivation evoked very specific changes in nesfatin level demonstrated by ISH and ELISA measurements, morphological studies were performed on hypothalamic sections of HC, SP and SPR animals. Rebound evoked a robust activation in nesfatin - positive cells in each investigated area (Fig. 3). In order to further characterize the nesfatin - producing cell population, we used triple fluorescent immunostaining for the visualization of nesfatin, MCH and Fos.

#### Co - localization of MHC and nesfatin

In home cage controls, most of the nesfatin-immunoreactive cells co - localized with MCH, with slight differences in the investigated areas (rate of MCH/nesfatin co - localization: ZI: 81.9±1.7%, perifornical area: 66.2±4.8%, LH: 75.4±2.1%, n = 5). The experimental conditions did not affect the percentage of nesfatin/MCH co - localization.

#### Neuronal activation due to the experimental conditions

Fos positivity in the HC and SP groups did not differ significantly from each other in any areas, although there was a difference between the MCH - negative and positive nesfatin populations. Nesfatin/MCH double positive neurons exhibited minimal activation (less than 0.5%). MCH - negative nesfatin neurons showed a substantial activity in the perifornical area, (24.4±8.1% and 39.4±7.4% in HC and SP groups, respectively), while in the ZI and the LH, the activity of the MCH - negative nesfatin neurons was less than 20% in both groups.

**Figure 4:**
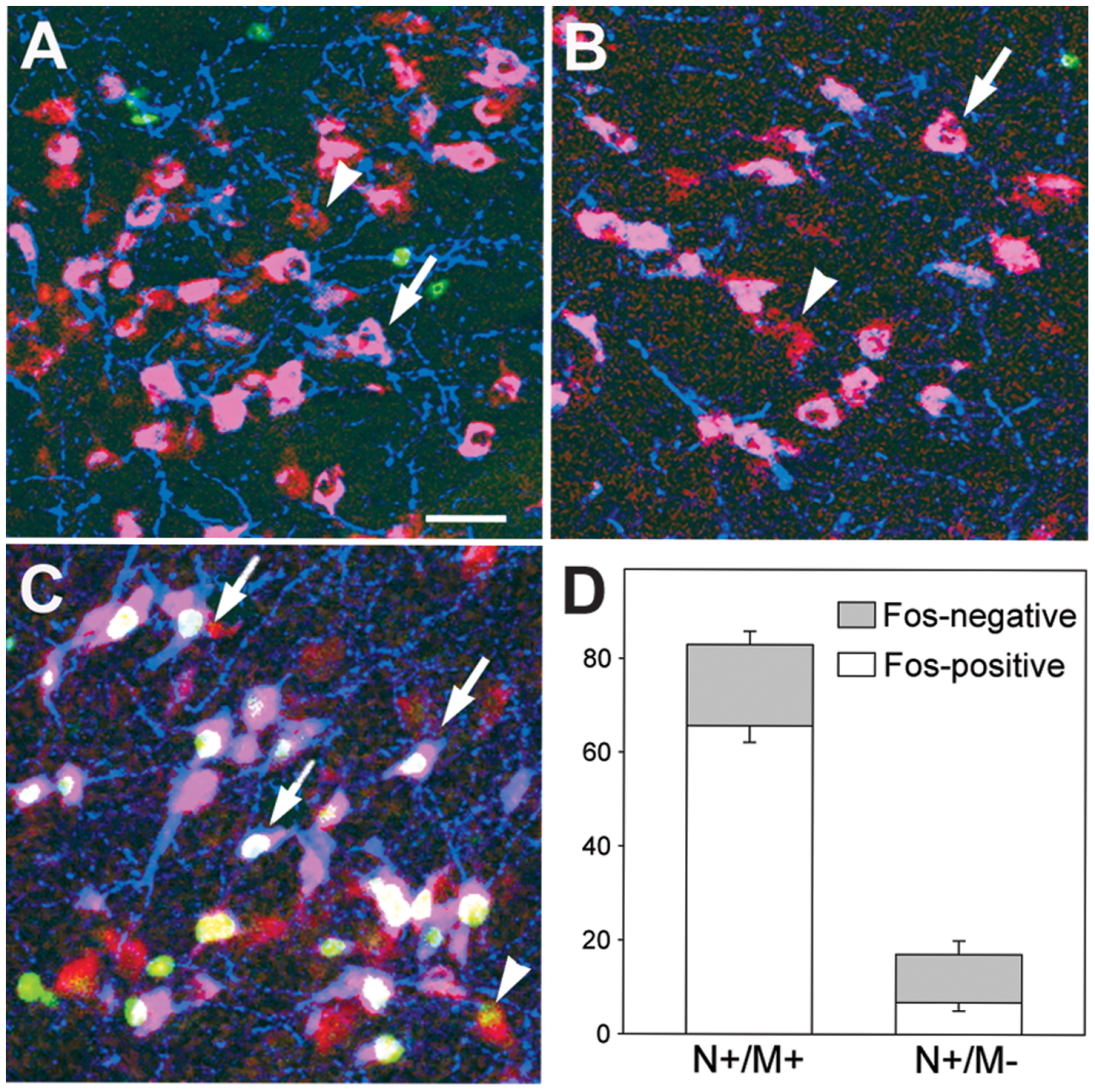
Participation of melanin-concentrating hormone (MCH)-positive and MCH - negative nesfatin-1/NUCB2 (nesfatin) neurons in the sleep - wake cycle. **A–C**. Illustrative pictures of the lateral hypothalamic area demonstrating the results of the triple fluorescent immunostainings for nesfatin (red), MCH (blue) and Fos (green) in a home cage kept (**A**), a rapid eye movement sleep (REMS) - deprived (**B**) and a REMS - deprived - sleep rebound (**C**) animal. Nesfatin/MCH double-positive neurons are pink (arrows), MCH - negative nesfatin neurons are red (arrowheads). Activated nesfatin/MCH neurons show white nuclei, activated nesfatin - positive, but MCH - negative neurons show yellow nuclei. Note that majority of the MCH - positive nesfatin neurons are activated (Fos - positive, arrows) by rebound, while only a few of the MCH - negative neurons showed Fos - positivity (arrowhead). Scale bar: 100 µm. **D**. Distribution of MCH - positive (N^+^/M^+^) and MCH - negative (N^+^/M^¯^) neurons within the nesfatin producing cell population and percentage of activated (Fos - positive) cells after REMS deprivation followed by rebound. Data are shown as mean ± SEM, n = 5.

Sleep rebound strongly activated the nesfatin/MCH double positive neurons regardless of the area examined (ZI: 86.9±2.9%, perifornical area: 78.3±2.7%, LH: 79.0±1.8%, Fig. 4). MCH - negative nesfatin neurons were not activated by rebound sleep in the ZI and the perifornical area, however, some activation was detected in the LH (Fig. 4C arrowhead), (SPR: 37.6±4.8%, p<0.01 *vs.* HC: 6.0±2.4% and SP: 15.0±3.1% groups, n = 5).

### Effects of icv Administered Nesfatin-1 on Vigilance from the 2^nd^ to the End of the 6^th^ Hours of Passive Phase

**Figure 5:**
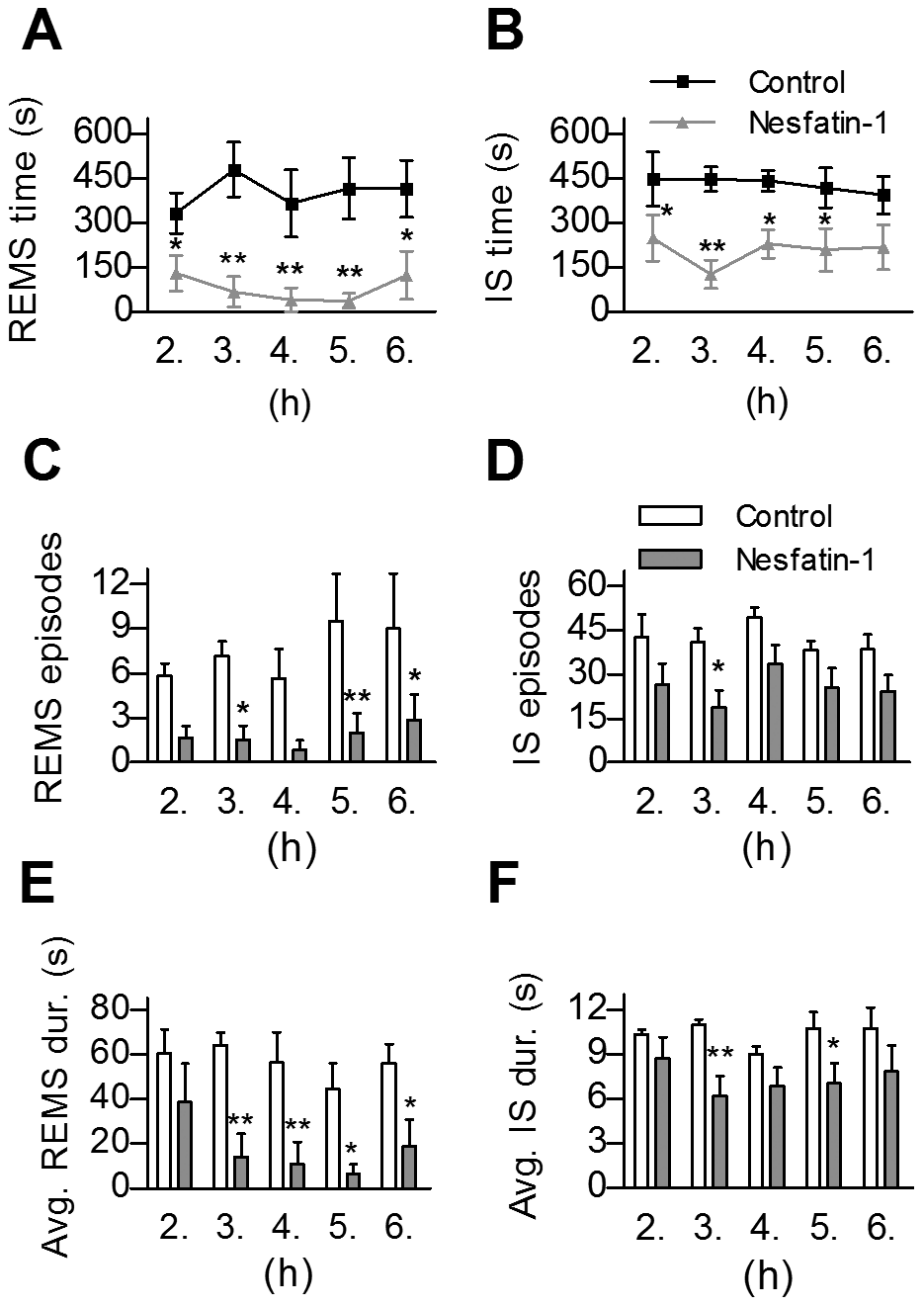
Effects of intracerebroventricularly administered nesfatin-1 on rapid eye movement sleep (REMS) and intermediate stage of sleep (IS). **A,B**. The time spent in REMS and IS per hour, respectively in the 2^nd^–6^th^ hours of passive (light) phase. **C,D**. The number and -E,F- the average duration of REMS and IS episodes per hour, respectively. Data are presented as mean ± SEM, n = 6 per group, p*<0.05, p**<0.01.

**Figure 6:**
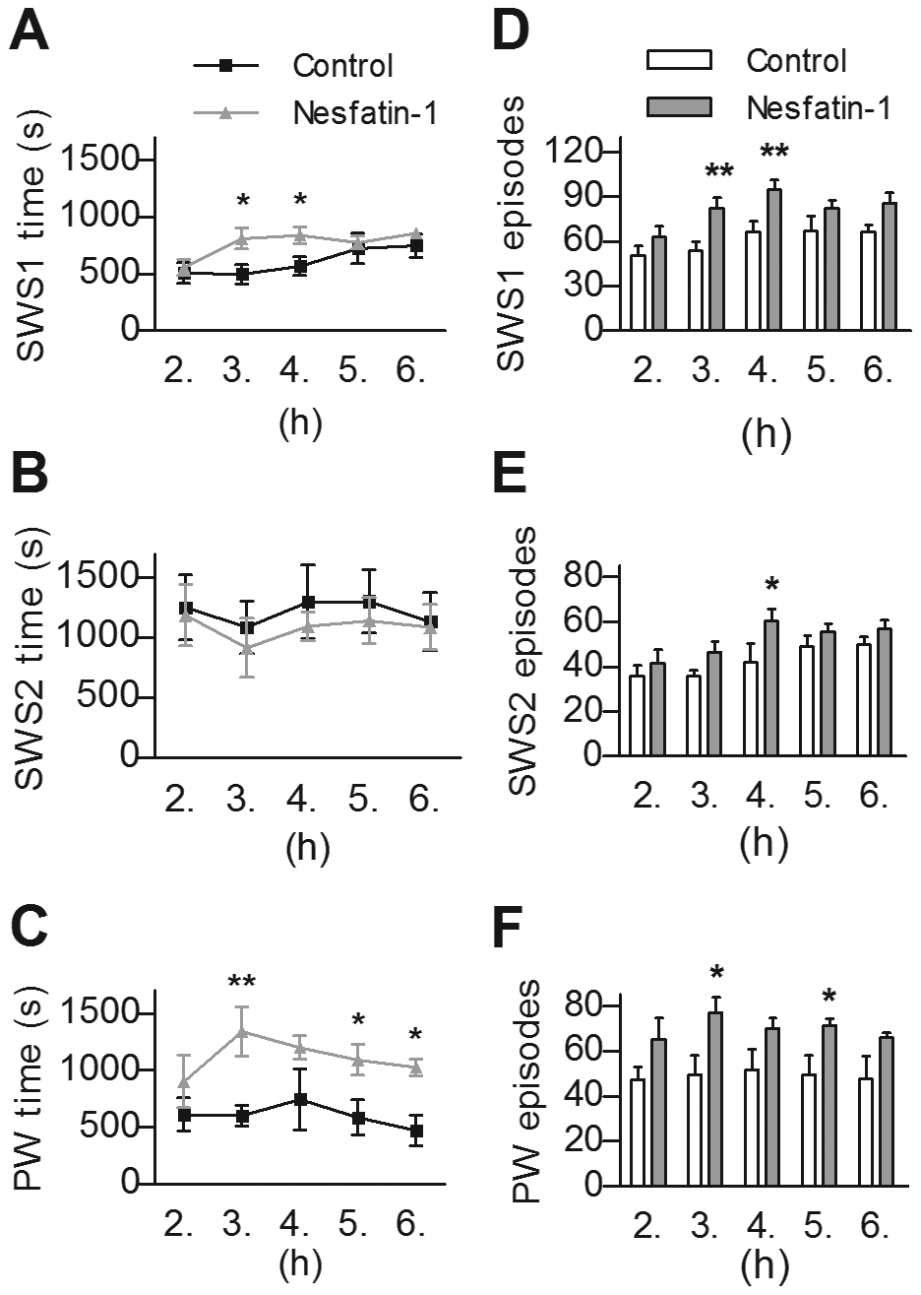
Effects of intracerebroventricularly administered nesfatin-1 on slow wave sleep and passive wake (PW) vigilance stages. The time spent and the number of episodes in light slow wave sleep (SWS1, **A** and **D**, respectively), deep slow wave sleep (SWS2, **B** and **E**, respectively) as well as in PW (**C** and **F**, respectively), per hour in the 2^nd^–6^th^ hours of passive (light) phase. Data are presented as mean ± SEM, n = 6 per group, p*<0.05, p**<0.01.

According to our results, icv administered nesfatin-1 significantly increased sleep fragmentation (F_(1,10)_ = 5.1046, p<0.05) and caused a trend-level decrease in total sleep time (p = 0.0587) during the five investigated hours of passive phase. Regarding the different sleep stages, nesfatin-1 markedly diminished the time spent in REMS (F_(1,10)_ = 18.99, p<0.001), compared to controls. This decrease was ca. 60% in the 2^nd^ h, however, in the next three hours of sleep, the fall in REMS time approached an approximate value of 90%, while in the 6^th^ h it was ca. 70% (Fig. 5A). Similarly, the amount of IS showed a significant decrease being the lowest in the 3^rd^ h (F_(1,10)_ = 11.04, p<0.01, Fig. 5B). The REMS and IS - declining effect in sleep time was also apparent in the number and the average duration of the episodes both in REMS (F_(1,10)_ = 10.45, p<0.01 and F_(1,10)_ = 12.81, p<0.01, respectively, Fig. 5C,E) and IS (F_(1,10)_ = 7.18, p<0.05 and F_(1,10)_ = 6.99, p<0.05, respectively, Fig. 5D,F). Considering the time spent in NREM sleep, a significant time × treatment interaction (F_(2,20)_ = 4.092, p<0.05) was revealed in SWS1, when repeated measure ANOVA was performed including the 2^nd^, 3^rd^ and 4^th^ hours only. The following *post hoc* test resulted in significant increase of SWS1 regarding the 3^rd^ and 4^th^ h (Fig. 6A). The time spent in SWS2 showed no alteration in any investigated hours (Fig. 6B).

The amount of PW elevated markedly (F_(1,10)_ = 8.955, p<0.05, Fig. 6C), in contrast to AW, which was unchanged (data not shown). Noteworthy, that the increase of SWS1 and PW was parallel to the elevation of episode-numbers generally (F_(1,10)_ = 9.87, p<0.05, Fig. 6D and (F_(1,10)_ = 6.46, p<0.05, Fig. 6F, respectively), although average episode-durations of these stages were unaffected. However, SWS2 showed no alteration despite an increase in the number of episodes in the 4^th^ h (Fig. 6E).

## Discussion

In this study we provide evidence for the implication of the recently identified anorexigenic molecule, nesfatin in the regulation of sleep of rat. According to our results, abolishment of REMS decreased nesfatin mRNA and protein expression in the DLH, a prominent site of nesfatin expression implicated in vigilance, feeding and depression [13], [18], [31], [32], while the subsequent REMS rebound restored these levels. We found that central administration of nesfatin-1 diminished the time spent in REMS and IS, while increased PW and SWS1, further reinforcing its impact in the regulation of vigilance. Very recently Jego et al. have also established the effect of icv nesfatin-1 on sleep architecture, although demonstrating different results [33]. The discrepancy may be explained by the different doses they used as well as the different timing of the icv nesfatin administration.

To abolish REMS completely, rats were kept on single platforms surrounded by water for 72 h [20], [21]. As muscle atony is typical of REMS, animals fall into the water and awake as they switch to REMS. Classic (one rat per cage on a single pot) and modified (several rats per cage on multiple pots) platform methods are widely used to abolish REMS [20], [21], [34], [35]. Multiple platforms were originally introduced as an alternate of single one to reduce stress caused by mobile restriction and social isolation [35]–[37]. However, even if rats are kept together from an early age to establish social stability and avoid stress caused by rearrangement of the social hierarchy when placed on multiple platforms, they show the same adrenocorticotropic hormone (ACTH), and just minimally higher corticosterone levels than rats on single platforms [38]. Because of these reasons, and since unique activity of MCH neurons during REMS recovery was demonstrated firstly with the classical flowerpot method [20], we adopted this technique.

To isolate effects of stress, LP kept animals were used as controls. The likewise elevated CRH levels in the hypothalamic paraventricular nucleus, the similar plasma ACTH and corticosterone concentrations [35] and the negative energy balance in both groups confirm that LP and SP animals are exposed to the same stress conditions with comparable activity of the hypothalamo - pituitary - adrenal axis. Another aspect of importance is thermogenesis due to wet fur. SP animals get wet when fall asleep, but LP animals also spend considerable amount of time in the water with swimming during the deprivation procedure. Thus, differences either in thermoregulation due to the wet fur and in the amount of the restraint stress, are not considered as major factors regarding the results detected only in SP animals. Furthermore, changes in the sleep architecture also designate large pot kept animals as useful controls. Both LP and SP rats have been reported to reduce SWS [37] in line with our paradigm, as some degree of SWS1 rebound was also observed in these groups [21]. The existence of REMS rebound in LPR animals and data of Machado et al. [21], [37] suggest that LP animals were subjected to REMS deprivation due to stress [39], but in the SP rats, REMS ceased totally, creating an elemental difference between LP and SP conditions [37]. This substantial contrast is reflected in the marked alterations of REMS architecture and connected Fos activity in the DLH of LPR rats, compared to SPR ones [21], [40]. As a consequence, any small pot - specific observation is likely due to the lack of REMS.

Our data suggest a striking link between nesfatin expression and REMS. Animals in the SP group reacted with an exclusive and remarkable decrease in nesfatin mRNA and protein levels to the lack of REMS, while nesfatin expression of animals in the HC and LP groups was alike. On the other hand, a significantly higher amount of REMS rebound with longer average duration of episodes compared to LPR and HCR groups [21] returned these values to the control levels. There was no difference between the HC and HCR groups indicating that circadian rhythm does not influence nesfatin expression in the first three hours of sleep after lights on.

Since nesfatin is an anorexigen and decrease of nesfatin mRNA level in certain hypothalamic nuclei, like the paraventricular and the supraoptic nuclei (but not in the DLH - unpublished observations), was established after fasting [41], [42], it is important to clarify whether negative energy balance of animals may, or may not be responsible for the reduction of nesfatin expression. It is noteworthy to mention that animals were not food - deprived during the 72 h on platforms. The cumulative food intake of rats was identical in all groups. In spite of this, both SP and LP animals had a decreased body weight, indicating elevated energy expenditure. However, they did not differ significantly in this respect, and there was no change in nesfatin mRNA expression in LP rats. Therefore, we can exclude the idea that the changes in the nesfatin expression in the SP animals may be related to the disturbed energy homeostasis.

To characterize both neurochemically and neuromorphologically the nesfatin - positive cell population responsible for the observed results, we applied multiple immunostainings. Majority of the nesfatin - expressing neurons co - expressed MCH, and practically all MCH cells co - expressed nesfatin, in agreement with earlier findings [10]. REMS rebound evoked Fos expression in large percent of nesfatin neurons. Most of these nesfatin neurons were MCH - positive confirming previous reports [20], [40], revealing a strong activation in all investigated areas. MCH - negative nesfatin cells showed little activation in the LH and no activation in both the ZI and the perifornical area. Since Fos is a transcription factor, indicating ongoing transcriptional activity [43], the appearance of Fos signal in the nesfatin - immunoreactive neurons is also in line with our findings showing an elevation of nesfatin mRNA and protein levels during REM rebound. The potential role of nesfatin in REMS regulation was further examined by analyzing the effect of the peptide administered centrally on vigilance stages in control rats. The applied 25 pmol dose was established according to previous studies [1], [4], [44]. As animals were presumably disturbed by the icv procedure, despite previous habituation [4], [21], moreover, increased plasma ACTH and corticosterone levels have been reported after icv nesfatin-1 injection in the first 30 and 60 min, respectively [4], [45], we omitted the first hour and evaluated vigilance from the beginning of the 2^nd^ to the end of the 6^th^ hours of passive phase.

Centrally injected nesfatin-1 had an IS-REMS decreasing effect lasting 5–6 hours following administration. Decrease in the number of REMS and IS as well as the average duration of REMS episodes seem to be involved in this effect, suggesting that neurons both in onset and maintenance of IS-REMS may be affected. The decline of REMS and IS was presumably compensated by a short-lived elevation of SWS1, and we also found a parallel increase in the amount of passive wake, and it was prolonged. There was a tendency for decrease of total sleep time.

The mechanism of nesfatin-1’s action on vigilance stages needs to be clarified in the future. Increase in the activity of the ascending arousal system and/or decrease in midbrain reticular arousal threshold by nesfatin-1 directly or indirectly, may be one explanation. Indeed, Yoshida et al. have reported that centrally administered nesfatin-1 induced Fos expression in noradrenergic neurons of the locus coeruleus and serotonergic cells of the dorsal and median raphe nuclei [45], structures known to be parts of the brainstem arousal system [46]. However, we should not ignore that icv nesfatin-1 has been demonstrated to cause Fos expression also in the PVN, the nucleus of the solitary tract and the nucleus supraopticus [45], all being related to stress and therefore, may potentially alter sleep architecture [39], [47], [48].

In relationship with MCH, it is interesting to notice a consequent opposite effect of nesfatin-1. Unlike nesfatin-1, MCH has been shown to induce a dose-dependent increase of REMS when centrally injected [20]. Ahnaou et al. have demonstrated that MCH_1_ receptor antagonist compounds decreased REMS, IS and SWS2, while wake stages increased [49]. Moreover, on food intake and energy expenditure, MCH and nesfatin act also oppositely [1], [15], [44]. Co - localization of MCH with other neuropeptides having opposite effect is not unique. A high percent of orexigenic MCH neurons co - localize with the anorexigenic cocaine and amphetamine regulated transcript [50].

Additionally, as for synaptic action of MCH, it has a predominantly inhibitory effect pre- and postsynaptically and attenuates the activation of N-, L- and P/Q-type calcium channels, while nesfatin-1 has been found to rise intracellular Ca^2+^ concentrations, by stimulating Ca^2+^ entry *via* N-, L- and P/Q-type calcium channels [12], [51]. At the same time, the receptor or receptors for nesfatin-1 have not been identified yet; therefore the precise mechanism of nesfatin’s action remains to be elucidated.

In summary, we revealed a close association between REMS architecture and nesfatin expression in the DLH and the ZI, as well as a possible negative feedback effect of nesfatin-1 on REMS, inasmuch as there is a disinhibition of nesfatin expression by REMS rebound in the DLH and ZI, and an inhibition of REMS by icv nesfatin-1. Further data are needed about the role of other nesfatin - expressing brain areas in this process that theoretically can be related to the sleep-wake regulation. Our results confirm earlier findings implicating the integrative role of the DLH and ZI in the control of a wide variety of functions, like energy homeostasis, vigilance, regulation of motor activity and reward, as well as more recently, in mood and depression [13], [14], [52], [53]. Sleep architecture of humans is strongly affected by several illnesses, like narcolepsy, anorexia and depression [7], [54]–[56]. Hence, the present findings may be relevant not only in sleep research, but also in other studies regarding sleep related disorders. Depression and anorexia are among those affecting the population worldwide. Thus, the possibility that nesfatin, the anorexigenic peptide, recently been related also to depression [5], [6] may emerge as a potential new link between these diseases and sleep disturbances, calls for further investigation.
